# Stochastic Dynamics of Gene Switching and Energy Dissipation for Gene Expression

**DOI:** 10.3389/fgene.2020.00676

**Published:** 2020-07-02

**Authors:** Quan Liu, FengZhen Yu, Liang Yi, Yijun Gao, Rong Gui, Ming Yi, Jianqiang Sun

**Affiliations:** ^1^Department of Physics, College of Science, Huazhong Agricultural University, Wuhan, China; ^2^Department of Chemistry, College of Science, Huazhong Agricultural University, Wuhan, China; ^3^College of Animal Science and Technology and College of Veterinary Medicine, Huazhong Agricultural University, Wuhan, China; ^4^School of Mathematics and Physics, China University of Geosciences, Wuhan, China; ^5^School of Automation and Electrical Engineering, Linyi University, Linyi, China

**Keywords:** non-equilibrium mechanisms, stochastic dynamics, gene switching, energy dissipation, chemical master equations

## Abstract

Stochastic dynamics of gene switching and energy dissipation for gene expression are largely unknown, mainly due to the complexity of non-equilibrium mechanisms. Here, based on an important double-deck loop model, the stochastic mechanisms of gene switching and energy dissipation are studied. First, the probability distributions of steady states are calculated theoretically. It is found that the signal can strengthen the choice of gene switching between the “off” and “on” states. Our analysis of energy consumption illustrates that, compared with the synthesis and degradation of proteins, the process of gene switching costs little energy. Our theoretical analysis reveals some interesting insights into the determination of cell state and energy dissipation for gene expression.

## 1. Introduction

Signal pathways play vital roles in life by cooperating to control more than one biochemical process while consuming free energy supplied by ATP or high-energy bonds to carry out different vital functions. Based on the core negative feedback control loop shared by various adaption biological systems, Lan et al. show that energy dissipation is indicated to stabilize the adapted state against noise (Lan et al., 2012). Further study explores the present analytic results on the non-equilibrium steady-state (NESS) of the model through mapping to a one-dimensional birth-death process, and the result suggests that the adaptation error can be reduced exponentially as the methylation range increases (Wang et al., 2015). In recent research, the number of phase coherent periods is found to be proportional to the free energy consumed per period (Cao et al., 2015). Increasingly numerous theoretical studies focus on the role of energy in biological information processes and biochemical signal transduction (Lan and Tu, 2013; Endres, 2017).

**Figure 1:**
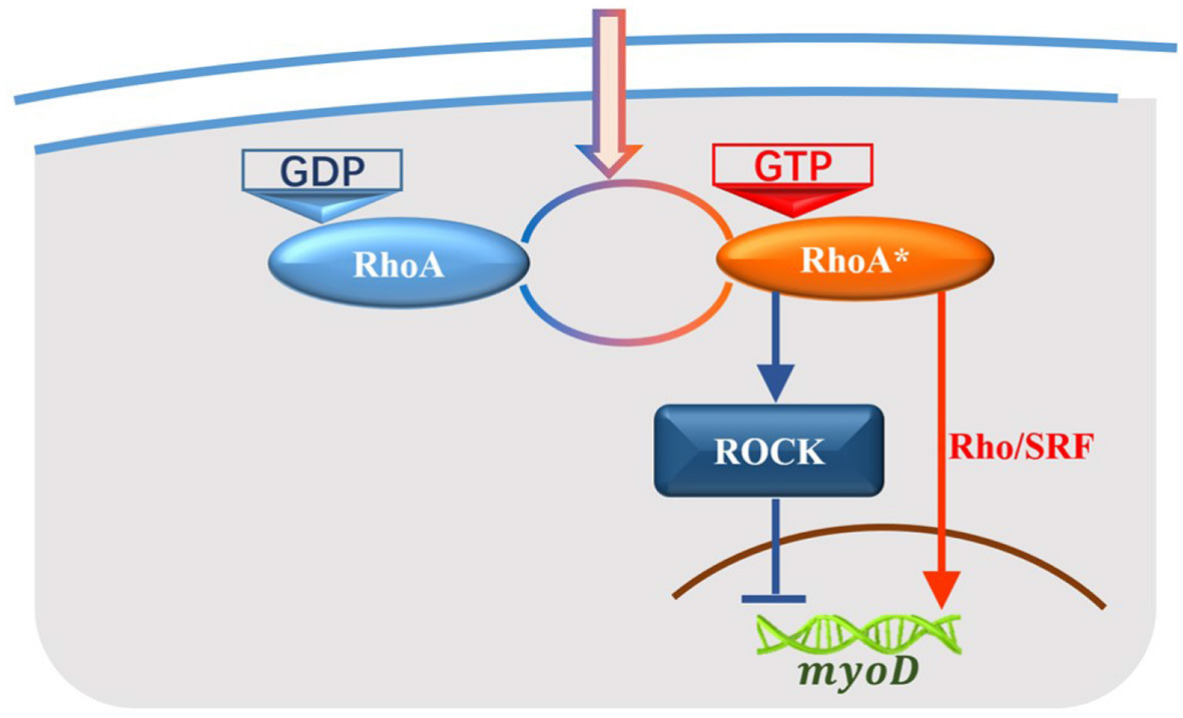
Signal pathways from RhoA to the target gene in the development of skeletal muscle. RhoA-GDP is activated by extracellular signals to RhoA-GTP (RhoA^*^) while RhoA^*^ is deactivated back to RhoA-GDP as well. RhoA^*^ contributes to the development of skeletal muscles through two signal pathways: RhoA/ROCK regulates the target gene *myoD* negatively which is corresponding to the blue cascade, and RhoA/SRF regulates *myoD* positively which is corresponding to the red cascade.

Biological information processes are complex. In the process of skeletal development, extracellular signals activate RhoA, and control the state of downstream genes mainly through two pathways: RhoA/SRF and RhoA/ROCK (Charrasse et al., 2002; Sordella et al., 2003; Tsai et al., 2013; Matsuoka and Yashiro, 2014). The marvelous phenomenon in this signal cascade is that those two pathways exert the opposite effects, as shown in Figure 1. Hence, such a signal cascade is composed of two competitive pathways and possesses the ability to accurately control the vital bio-processes (Wei et al., 1998, 2000, 2001; Meriane et al., 2000; Beqaj et al., 2002; Castellani et al., 2006; Charrasse et al., 2006). The study of stochastic dynamics and energy dissipation in this biological system represents an interesting topic. The competitive networks may present two kinetic characteristics: oscillation and bistable state. Ouyang et al. show a series of works on the non-equilibrium thermodynamics of oscillations within cells and their results revealed that the critical energy dissipation per period depends on both the frequency and strength of the exchange reaction which gave an optimal design for achieving maximum synchronization with a fixed energy budget (Cao et al., 2015; Fei et al., 2018; Zhang et al., 2020). Gene switch as another kind of competitive networks is a representative bistable state system and will be the focus of our study.

**Figure 2:**
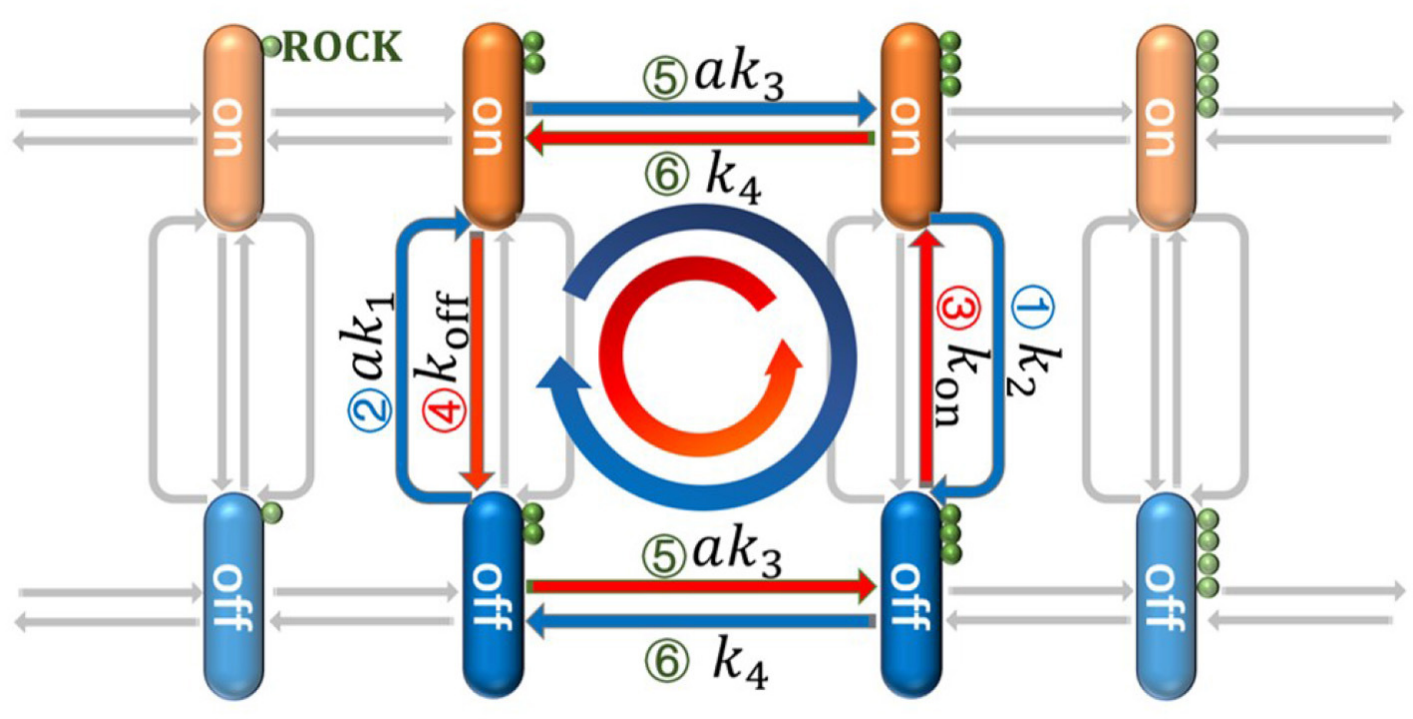
The detailed cascades in the double-deck loop (DDL) which is a simplified model based on the biochemical reaction networks in Figure 1. The orange stick is the gene in the “on” state and the blue one is the gene in the “off” state; ROCK is represented as the small green ball; Arrow 1 corresponds to the Rho/ROCK pathway in Figure 1 and Arrow 2 corresponds to the Rho/SRF pathway. The negative regulation from ROCK can push down the state of gene from the “on” state to the “off” state along Arrow 1 with rate *k*_2_. The positive regulation from RhoA^*^ can also push up the state of gene along Arrow 2 with rate *ak*_1_. The Arrow 3 and 4 represent the basic switching of gene's state with rates *k*_on_ and *k*_off_ respectively. The synthesis and degradation of ROCK will drive the system move along the horizontal directions (i.e., Arrow 5 and 6) with rates *ak*_3_ and *k*_4_, respectively. Since two parallel loops (i.e., the blue and red loops) can be found in our model, it is so called DDL.

In this paper, we propose a double-deck loop (DDL) model to describe signal cascades which are similar to those found in the development of skeletal muscle, as shown in Figure 2. By virtue of non-equilibrium statistical physics theory and stochastic dynamics, exact analytical solutions to the steady-state probability distribution are obtained and energy dissipation in the DDL is derived, which allows for many deeper discussions. Our aims are to reveal (i) the crucial factors that determine the state of gene switching in our model and (ii) the energy dissipation in biochemical reactions. We expect that these theoretical results could help us to understand the general principles of signaling selectivity and energy dissipation in gene regulation networks.

## 2. The Model

In the process of skeletal development, RhoA is activated by extracellular signals from RhoA-GDP to RhoA-GTP (RhoA^⋆^), and RhoA^⋆^ is deactivated back to RhoA-GDP as well. RhoA^⋆^ plays a key role in process of skeletal development and contributes to the regulation of the expression of muscle-specific genes both through RhoA/SRF and RhoA/ROCK signal pathways. RhoA/ROCK pathway triggers a negative control function on the target gene while RhoA/SRF pathway induces a positive regulation. The relevant biochemical processes are shown in Figure 1. The red signal cascade represents RhoA/SRF pathway and the blue one represents RhoA/Rock pathway.

To derive our mathematical model, some assumptions are put forward. (i) The switching dynamics between GTP and GDP have been discussed thoroughly (Lan and Tu, 2013) which has the same dynamic mechanism with the switching between RhoA^⋆^ and RhoA-GDP. This aspect is neglected in our model, since we focus on the selectivity between different gene's state modes and energy dissipation of RhoA/ROCK and RhoA/SRF signal pathways. These two pathways are adopted to regulate the development of skeletal muscle. (ii) It is well-known that a large number of genes are involved in this biological process (Matsui et al., 1996). If all genes are considered, it will complicate the modeling and theoretical analysis. We hypothesize that the development state of skeletal muscle can be represented with the state of gene *myoD*. Muscle-specific genes begin to be expressed when the state of gene *myoD* is “on”, otherwise these genes are closed when the state of gene *myoD* is “off”. (iii) Since the role of ROCK in biological activities is vital (Leung et al., 1995; Aelst and D'Souza-Schorey, 1997; Kaibuchi et al., 1999; Cloutier et al., 2010), the detailed biochemical process of RhoA/SRF pathway is neglected and the regulation of RhoA/SRF pathway is simplified to the direct regulation of RhoA^*^ on gene *myoD* as shown in Figure 1. Therefore, we mainly aim to discuss the biochemical reactions including the switch of *myoD* state and synthesis/degradation of ROCK. Based on the above assumptions, the detailed biochemical equations are as follows:

(1) $$\begin{array}{l} \;{\text{RhoA}}^{*}\overset{k_{3}}{\to }{\text{RhoA}}^{*}+\text{ROCK},\text{ROCK}\overset{k_{4}}{\to }\emptyset \\ G_{\text{off}}+{\text{RhoA}}^{*}\overset{k_{1}}{\to }G_{\text{on}}+{\text{RhoA}}^{*},G_{\text{off}}\overset{k_{\text{on}}}{\to }G_{\text{on}}\\ G_{\text{on}}+\text{ROCK}\overset{k_{2}}{\to }G_{\text{off}}+\text{ROCK},G_{\text{on}}\overset{k_{\text{off}}}{\to }G_{\text{off}}. \end{array}$$

The “on” state of *myoD* is indicated as *G*_on_ in Equation (1), while the “off” one is *G*_off_. *k*_1_ is the transition rate of gene state under the positive control and *k*_2_ is the transition rate of gene under the negative control. The basic switching rates between *G*_on_ and *G*_off_ are *k*_off_ and *k*_on_. *k*_3_ is the synthesis rate of ROCK and *k*_4_ is the degradation rate of it.

As shown in Equation (1), every state of gene can be achieved through two ways: *G*_on_ can be achieved both through the promotion of RhoA^*^ and the basic switching; *G*_off_ can be achieved both through the repressive control of ROCK and the basic switching. Considering the synthesis and degradation of ROCK which correspond to the increasing and decreasing of the small green balls in Figure 2, two parallel loops can be found. One is clockwise [i.e., $G_{\text{on}}\left(m\right)\overset{ak_{3}}{\to }G_{\text{on}}\left(m+1\right)\overset{k_{2}}{\to }G_{\text{off}}\left(m+1\right)\overset{k_{4}}{\to }G_{\text{off}}\left(m\right)\overset{ak_{1}}{\to }G_{\text{on}}\left(m\right)$] which is represented as a blue loop in Figure 2. The other one is anticlockwise [i.e., $G_{\text{on}}\left(m\right)\overset{k_{\text{off}}}{\to }G_{\text{off}}\left(m\right)\overset{ak_{3}}{\to }G_{\text{off}}\left(m+1\right)\overset{k_{\text{on}}}{\to }G_{\text{on}}\left(m+1\right)\overset{k_{4}}{\to }G_{\text{on}}\left(m\right)$] which is represented as red loop in Figure 2. Since these two loops are two parallel decks between *G*_on_ and *G*_off_, this theoretical model is called “double-deck loop (DDL)” in this paper. Moreover, *m* is the number of ROCK, and *a* is the concentration of RhoA^*^. Based on above biochemical equations, the chemical master equations (i.e., CME) can be presented as

(2) $$\begin{array}{l} \frac{dP_{0}\left(m,t\right)}{dt}=\;k_{\text{off}}P_{1}\left(m,t\right)-k_{\text{on}}P_{0}\left(m,t\right)+mk_{2}P_{1}\left(m,t\right)\\ \;-ak_{1}P_{0}\left(m,t\right)+ak_{3}P_{0}\left(m-1,t\right)-ak_{3}P_{0}\left(m,t\right)\\ \;+\left(m+1\right)k_{4}P_{0}\left(m+1,t\right)-mk_{4}P_{0}\left(m,t\right);\\ \frac{dP_{1}\left(m,t\right)}{dt}=\;-k_{\text{off}}P_{1}\left(m,t\right)+k_{\text{on}}P_{0}\left(m,t\right)-mk_{2}P_{1}\left(m,t\right)\\ \;+ak_{1}P_{0}\left(m,t\right)+ak_{3}P_{1}\left(m-1,t\right)-ak_{3}P_{1}\left(m,t\right)\\ \;+\left(m+1\right)k_{4}P_{1}\left(m+1,t\right)-mk_{4}P_{1}\left(m,t\right) \end{array}$$

where *P*_0_(*m, t*) is the probability of the gene “off” state and *P*_1_(*m, t*) are the probability of the gene “on” states.

## 3. Results

### 3.1. The Analytical Solutions of the DDL Model

Regulatory networks generally consist of interactional signal pathways. Different signal pathways may dominate cell fate in different circumstances. Based on the biology processes in the development of skeletal (Wei et al., 1998, 2000, 2001; Meriane et al., 2000; Beqaj et al., 2002; Castellani et al., 2006; Charrasse et al., 2006), the DDL model has both activation and inhibitory signal pathways originating from the same input. Therefore, it is interesting to identify the crucial factors in the selection of different signaling pathways. To address this problem, the probability distributions under different *a* are derived. In response to a persistent input, the synthesis and degradation of ROCK induce the evolution of ROCK, resulting in a steady state of the number of ROCK *m*. Using the method of probability-generating functions (Qian, 2007; Huang et al., 2015), the analytical expressions of steady-state probability distributions *P*_0_(*m*) and *P*_1_(*m*) are obtained

(3) P0(m)=1kon+ak1(ak2k3k2+1+koff)A01m!(ak3k2+1)m             ×∑l=0mk2lCml(α)l(β)lF11(α+l,β+l;ω2)             -1kon+ak1ak2k3k2+1αβA01m!(ak3k2+1)m             ×∑l=0mk2lCml(α+1)l(β+1)lF11(α+1+l,β+1+l;ω2)             +ak2k3kon+ak1αβA01(m-1)!(ak3k2+1)m-1             ×∑l=0m-1k2lCm-1l(α+1)l(β+1)lF11(α+1+l,β+1+l;ω2);P1(m)=A01m!(ak3k2+1)m             ×∑l=0mk2lCml(α)l(β)lF11(α+l,β+l;ω2)

where $\alpha =ak_{2}k_{3}/{\left(k_{2}+1\right)}^{2}+\left(k_{\text{on}}+k_{\text{off}}+ak_{1}\right)/\left(k_{2}+1\right)$,β = α + 1, and $\omega_{2}=-ak_{2}k_{3}/{\left(k_{2}+1\right)}^{2}$. Here, all the parameters are normalized by *k*_4_, i.e., *k*_*i*_ = *k*_*i*_/*k*_4_ which is different with Equation (2). $C_{m}^{l}$ is the binomial coefficient in Equation (3), (γ)_*l*_ is the Pochhammer symbol defined as (γ)_*l*_ = Γ(γ + *l*)/Γ(γ) with Γ(γ) being the Gamma function, and _1_*F*_1_(α, β; ω_2_) is a confluent hypergeometric function (Huang et al., 2015). *A*_0_ is the normalization constant as follows

(4) A0−1=eak3k2+1[1kon+ak1(ak2k3k2+1+koff)F11(α,β;ω1)        −1kon+ak1ak2k3k2+1αβF11(α+1,β+1;ω1)       +ak2k3kon+ak1αβF11(α+1,β+1;ω1)]+eak3k2+1F11(α,β;ω1)

where ω_1_ is a constant with the expression $\omega_{1}=ak_{2}k_{3}/\left(k_{2}+1\right)-ak_{2}k_{3}/{\left(k_{2}+1\right)}^{2}$. The details of analysis for chemical master equations are presented in the Supplementary Material of this paper. The above results will be checked through the structure of our model in the following part.

The character of signaling cascades in the development of skeletal muscle can provide us with information to verify our analytical solutions. The changes of ROCK, as an upstream component of the signaling cascades, follow a basic process of birth and death. The statistical law of a birth and death process is that the probability distribution about *m* is a standard Poisson distribution. In order to test this, we calculate the total probability *P*(*m*) which is provided by *P*_0_(*m*) + *P*_1_(*m*). This represents the statistical law of ROCK and can be simplified from Equation (3) as:

(5) $$\begin{array}{l} P\left(m\right)=\frac{1}{m!}{\left(ak_{3}\right)}^{m}e^{-ak_{3}}. \end{array}$$

**Figure 3:**
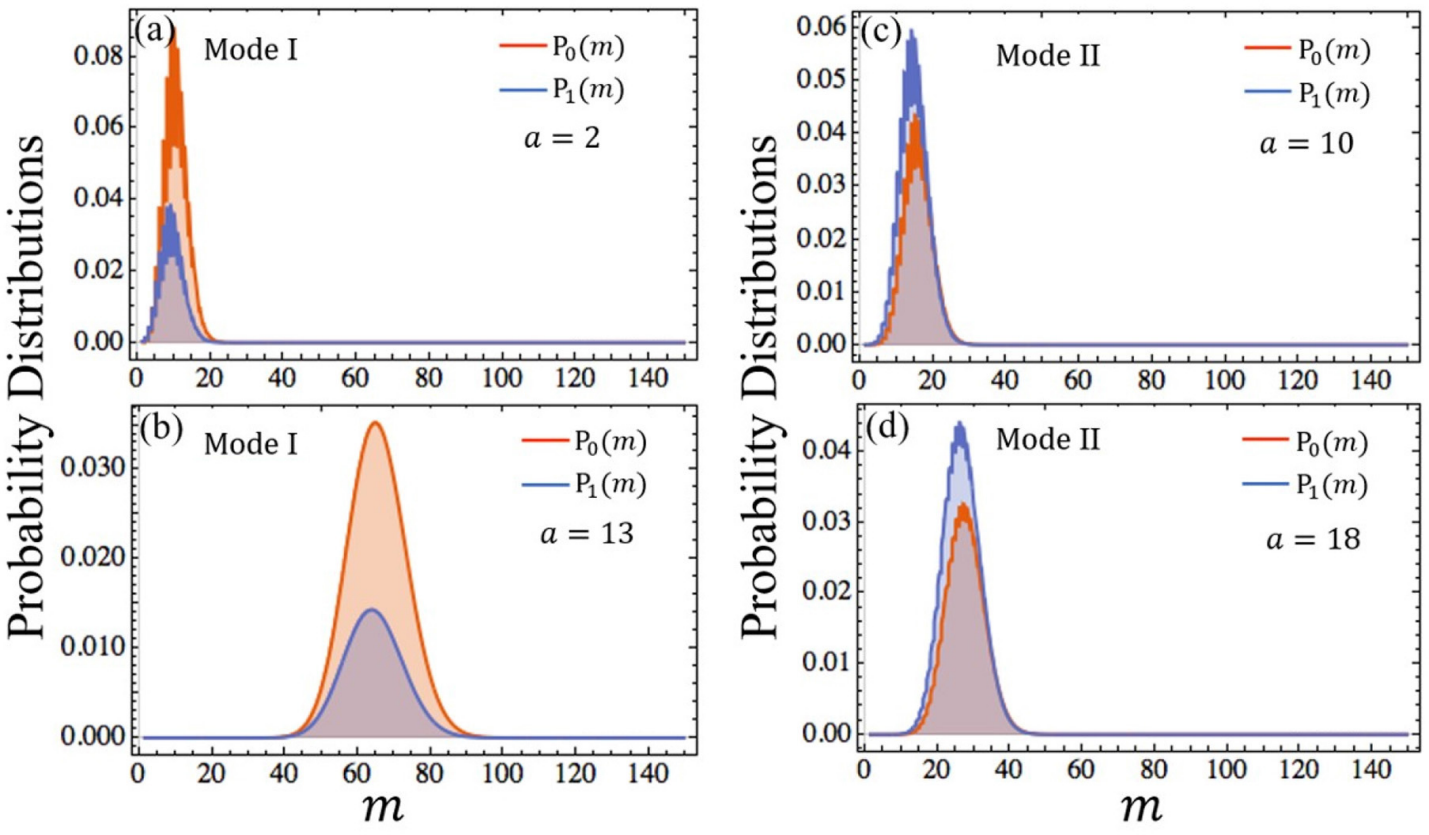
Distributions of probability as a function of *m*. The synthetic rate of ROCK *k*_3_ = 5 in **(a,b)** and *k*_3_ = 1.5 in **(c,d)**. The value of other parameters can be found in Table S1. *P*_0_(*m*) is the probability of the gene “off” state indicated with orange curve, *P*_1_(*m*) is the probability of gene “on” state indicated with blue curve.

This is a standard Poisson distribution. Furthermore, ROCK should be in its steady state *m*_*s*_ = *ak*_3_ most of the time. This means that the peaks of *P*_0_(*m*) and *P*_1_(*m*) focus on *m*_*s*_. According to Equation (3), the values of *P*_0_(*m*) and *P*_1_(*m*) with different parameters are computed through “Mathematica” which are shown in Figure 3. It is obvious that the peaks of distributions occur at the steady state value of *m*_*s*_. It's worth mentioning that the roughness of the curves shown in Figures 3a,c,d is caused by the computational accuracy of “Mathematica” when it is used to calculate the confluent hypergeometric function rather than biological or physical factors. We can also get these curves through “Matlab” which appear very smooth. However, compared with “Mathematica”, “Matlab” fails to calculate confluent hypergeometric function when *m* is too large. In order to verify these curves, the corresponding curves with the same parameters obtained by Monte Carlo simulation are shown in Figures S1a,b. It is obviously that the curves in Figures S1a,b closely resemble the ones in Figures 3a,b, respectively.

The above discussion confirms the reliability of our theoretical results in Equation (3). Based on those results, we will try to explore the selectivity of different pathways and energy consumption in the following section.

### 3.2. Stochastic Dynamics of Gene Switching in DDL

**Figure 4:**
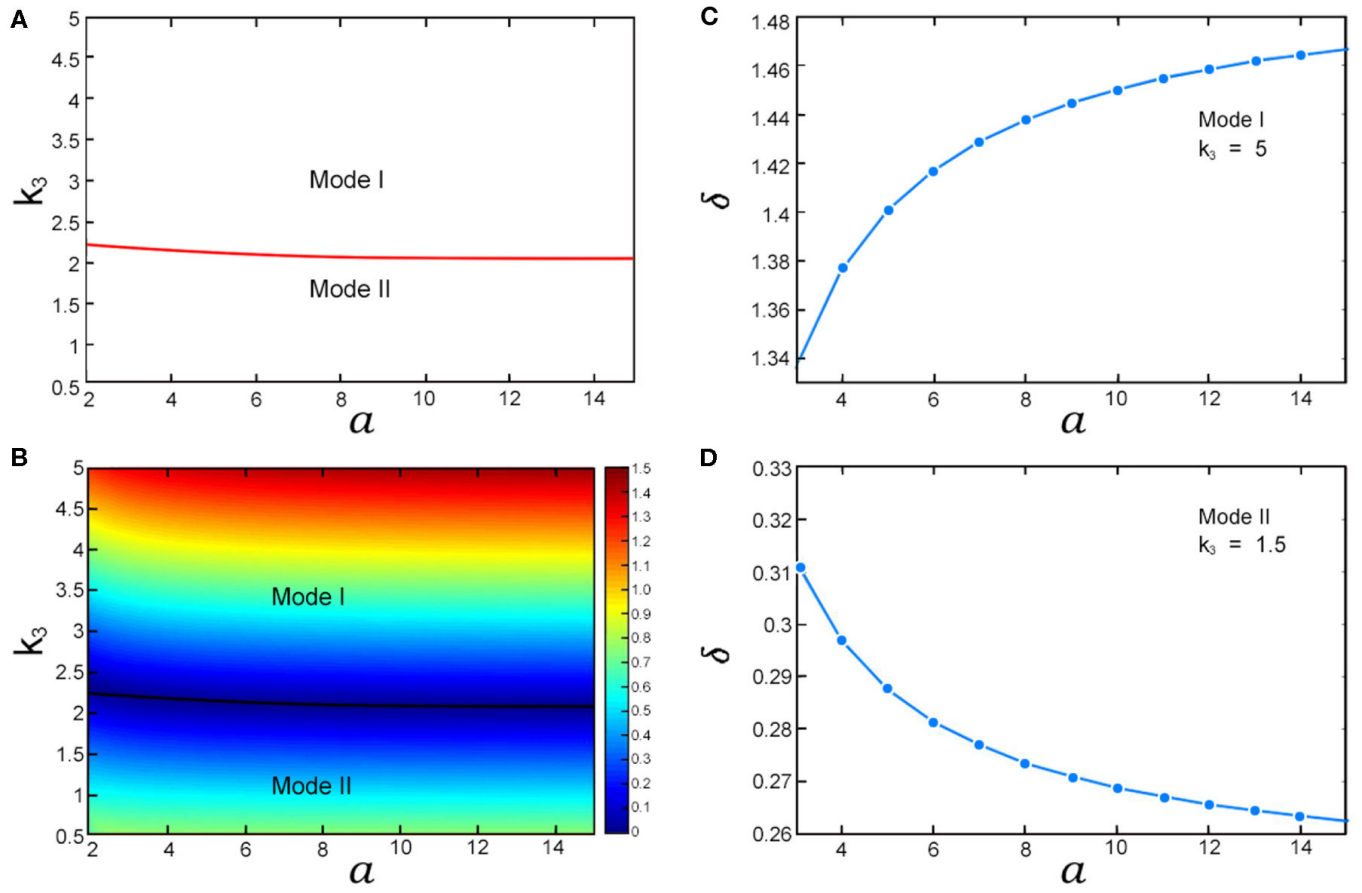
The influences of *k*_3_ and *a* on gene's “off” and “on” states. **(A)** The areas of Mode I and Mode II in *a*-*k*_3_ plane; **(B)** The heat map of gene's state dominance factor δ in *a*-*k*_3_ plane. The black line is the boundary between Mode I and Mode II; **(C)** The curve between δ and *a* in Mode I; **(D)** The curve between δ and *a* in Mode II. The values of other parameters are listed in Table S1.

The steady-state probability distributions with different stimulation strength are displayed in Figure 3. $\sum_{m}P_{0}\left(m\right)$ and $\sum_{m}P_{1}\left(m\right)$ which correspond to the areas under the curves of *P*_0_(*m*) and *P*1(*m*) are respectively the probabilities of the gene's “off” state and “on” state. We use them to define two gene modes: Mode I and Mode II. Mode I denotes that the probability of the gene's “off” state is larger than the one of the “on” state, and Mode II denotes that the probability of the gene 's “on” state is larger than the one of the “off” state. As shown in Figures 3a,b, when *k*_3_ = 5, the gene's state is Mode I. Conversely, if *k*_3_ = 1.5, the gene's state becomes Mode II shown in Figures 3c,d. Compared with *k*_3_, even if the parameter *a* varies widely, the gene's state mode is not changed. Figure 4A shows the areas of these two modes. It is obviously that the boundary between them is almost a horizontal line where *k*_3_ ≈ 2.1. This means that the mode of the gene's state is determined primarily by *k*_3_ and the parameter *a* has little effect on the selection of gene's state modes. Since *k*_3_ and *a* represent the synthesis rate of the negative controller ROCK and the strength of external stimulations, respectively, the gene's state mode depends almost exclusively on the synthesis rate of the negative controller ROCK rather than the external stimulations.

By the definition of Mode I, the gene is more likely to be in the “off” state than the “on” state. In other words, the ‘off” state of gene is dominant in Mode I. Similarly, the “on” state of gene is dominant in Mode II. To quantify the dominance of the gene's state, we define a gene's state dominance factor δ = |*P*_0,*max*_ − *P*_1,*max*_|/*P*_1,*max*_. The larger peak in *P*_*i*_(*m*) (*i* = 1, 2) means more dominance as shown in Figure 3. Therefore, δ can be used to measure the dominance of the gene's state. Next, we will discuss the influences of the synthesis rate of the negative controller ROCK and the external stimulations on gene's state dominance. The values of δ in *a*-*k*_3_ plane correspond to the scale values of the color bar in Figure 4B. As shown in Figure 4B, with increasing *k*_3_ (i.e., the synthesis rate of the negative controller ROCK), δ (i.e., the dominance of the gene's “off” state) increases in Mode I. Conversely, as *k*_3_ increases, δ decreases in Mode II. That suggests that when the synthesis rate of the negative controller ROCK increases, the dominance of the gene's “off” state is gradually weakening in Mode II, until the mode of the gene's state changes to Mode I and the dominance of the gene's “on” state increases gradually. Compared with *k*_3_, the influence of the strength of external stimulations RhoA^*^ (i.e., *a*) on the dominance of the gene's state (i.e., δ) is not obvious enough in Figure 4B. Therefore, the relation between *a* and δ with different parameters *k*_3_ which correspond respectively to Mode I and Mode II is shown in Figures 4C,D (Note that we also use Monte Carlo simulation to verify the trend of δ with *a* in Mode I which is shown in Figure S2). Similar to the case of *k*_3_, with increasing the strength of external stimulations RhoA^*^ (i.e., *a*), the dominance of the gene's state (i.e., δ) increases in Mode I and decreases in Mode II. That means that although external stimulations RhoA^*^ has little effect on the selection of gene's state modes, it can fine-tune the dominance of the gene's state in its respective modes.

In summary, the core factor for the stochastic dynamics of gene switching in DDL-type biochemical networks is identified in our work. Reaction rates are responsible for the selectivity between different gene's states, while the external signal stimulation fine-tunes the choice in its respective modes. The cooperation between signals and network maintains the vital process in an orderly manner.

### 3.3. Energy Dissipation in DDL

It is intuitively obvious that living biochemical systems need free energy (Gui et al., 2016, 2018). From the viewpoint of thermodynamics, gene expression is essentially a non-equilibrium process due to feedback or feedforward regulation that breaks detailed balance and thus necessarily consumes energy (Lu et al., 2017). But how is energy actually utilized during the regulation of gene expression in the development of skeletal muscle? To our knowledge, few works have touched upon this point. The composition of total energy consumption may help us grasp the selection mechanism between different biochemical processes.

From the definition of entropy $S\left(P_{i}\right)=k_{B}T\sum_{i}P_{i}\ln P_{i}$, the entropy production rate ε_*p*_(*t*) is given as follows (Ge and Qian, 2010):

(6) $$\begin{array}{l} \varepsilon_{P}\left(t\right)=\underset{i,j}{\sum }\left(P_{i}\left(t\right)q_{ij}-P_{j}\left(t\right)q_{ji}\right)\ln\left(\frac{q_{ij}}{q_{ji}}\right). \end{array}$$

*P*_*i*_(*t*) is the probability of the system in state *i* at time *t*, while *q*_*ji*_ is the transport rate from state *j* to *i*. *k*_*B*_*T* is set to be 1 for convenience in our work. ε_*p*_(*t*) is the sum of energy dissipated in the biochemical network. Furthermore, the entropy production rate for a non-equilibrium steady-state system can be calculated as

(7) $$\begin{array}{l} EP=\underset{\left(\sigma ,\sigma^{\prime }\right)}{\sum }P\left(\sigma \right)k\left(\sigma ,\sigma^{\prime }\right)\log\left(\frac{k\left(\sigma ,\sigma^{\prime }\right)}{k\left(\sigma^{\prime },\sigma \right)}\right) \end{array}$$

where *k*(σ, σ′) is the transition probability from state σ to σ′.

Considering the detailed biochemical reactions in our model, we derive the *EP* of the DDL network as

(8) $$\begin{array}{l} EP=\sum_{m}[P_{0}(m)ak_{3}\ln\left(\frac{ak_{3}}{m+1}\right)+P_{0}(m)m\ln\left(\frac{m}{ak_{3}}\right)\\ \;+P_{0}(m)(ak_{1}+k_{\text{on}})\ln\left(\frac{ak_{1}+k_{\text{on}}}{k_{\text{off}}+mk_{2}}\right)\\ \;+P_{1}(m)ak_{3}\ln\left(\frac{ak_{3}}{m+1}\right)+P_{1}(m)m\ln\left(\frac{m}{ak_{3}}\right)\\ \;+P_{1}(m)(k_{\text{off}}+mk_{2})\ln\left(\frac{k_{\text{off}}+mk_{2}}{ak_{1}+k_{\text{on}}}\right)]. \end{array}$$

**Figure 5:**
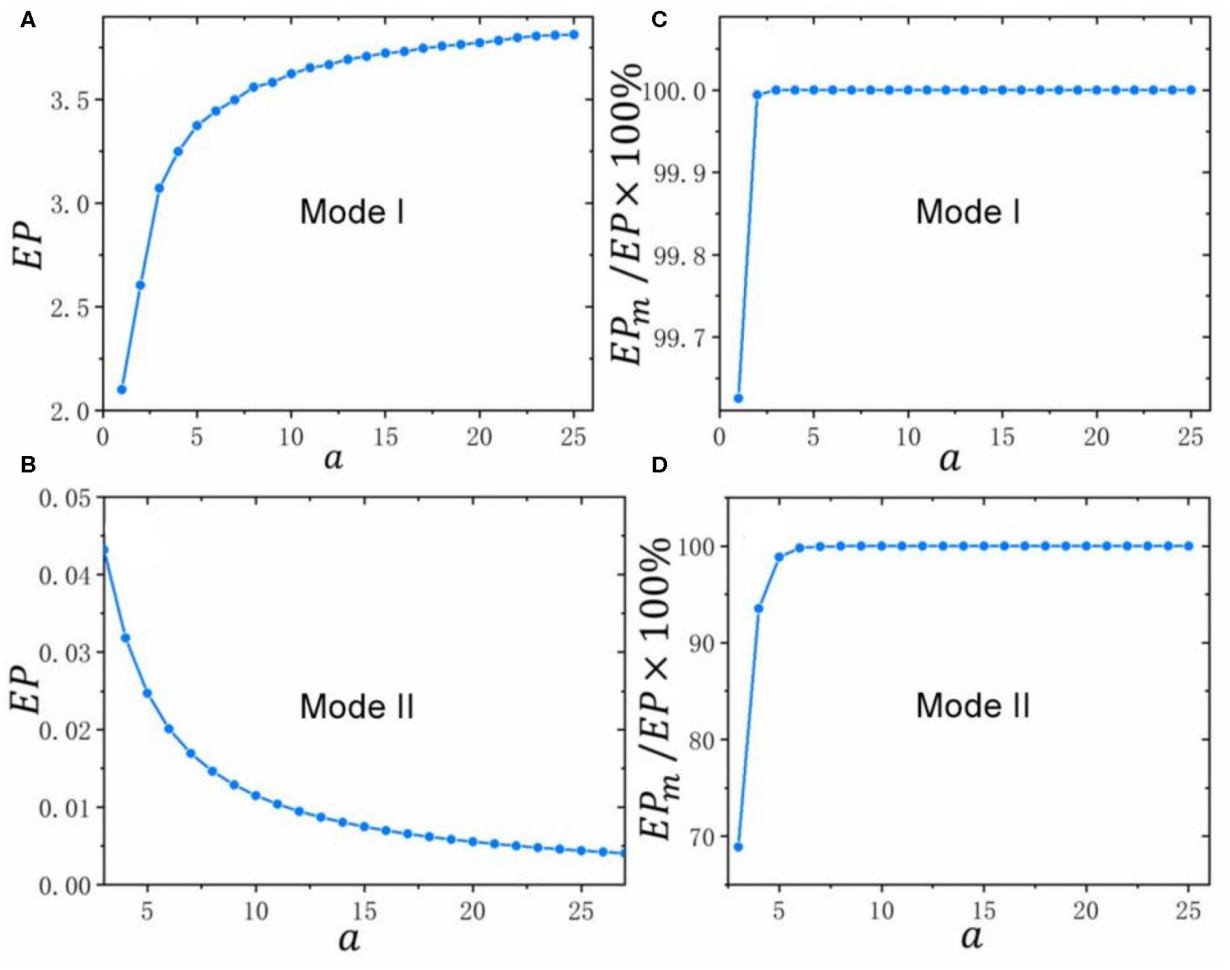
The influences of the strength of external stimulation on energy dissipation in Mode I and II. The values of all parameters are listed in Table S1. **(A,B)** The relation curves between total energy dissipation *EP* and *a* in Mode I and II; **(C,D)** The relation curves between the percentages of energy dissipation in the synthesis-degradation process of ROCK (i.e., *EP*_*m*_) in total energy dissipation (i.e., *EP*) and *a* in Mode I and II.

Next, we discuss the influences of the strength of external stimulation on the total energy dissipation *EP* in Mode I and Mode II respectively. As shown in Figure 5A, *EP* increases and its rate of increase decreases with increasing *a* in Mode I. This suggests that when the strength of external stimulation increases, the system consumes more and more energy to response it. Compared with the small strengthen of external stimulation, there is less growth of energy for the system corresponding to the large one in Mode I. In contrast to Mode I, both *EP* and its reduction rate decrease in Mode II as *a* increases (Figure 5B). This means although the system requires less and less energy with the increase of the strengthen of external stimulation, a small amount of energy is still needed to sustain it in Mode II.

Since the control of *MyoD* gene expression can be divided into two parts: one is the synthesis and degradation of ROCK and the second is the switching of gene state, the process of energy dissipation can be decomposed into three state transitions: (*m* − 1, off) ⇌ (*m*, off) ⇌ (*m* + 1, off), (*m* − 1, on) ⇌ (*m*, on) ⇌ (*m* + 1, on) and (*m*, on) ⇌ (*m*, off). Here, (*m*, on) represents the state when the gene is “on” and the level of ROCK is *m*. Note that the first two formulas are related to the synthesis and degradation of ROCK. According to these three state transitions, the total energy dissipation *EP* is decomposed into three terms as follows:

(9) $$\begin{array}{l} \begin{array}{l} EP_{1}=\sum_{m}[P_{0}(m)ak_{3}\log\left(\frac{ak_{3}}{m+1}\right)+P_{0}(m)m\log\left(\frac{m}{ak_{3}}\right)] \end{array}\\ \begin{array}{l} EP_{2}=\sum_{m}[P_{1}(m)ak_{3}\log\left(\frac{ak_{3}}{m+1}\right)+P_{1}(m)m\log\left(\frac{m}{ak_{3}}\right)] \end{array}\\ \begin{array}{l} \begin{array}{l} EP_{3}=\sum_{m}[P_{0}(m)ak_{1}+k_{on}\log\left(\frac{ak_{3}+k_{on}}{k_{\text{off}}+mk_{2}}\right)\\ \;+P_{1}(m)(k_{\text{off}}+mk_{2})\log\left(\frac{k_{\text{off}}+mk_{2}}{ak_{1}+k_{on}}\right)]. \end{array} \end{array} \end{array}$$

Since the synthetic rate of ROCK *k*_3_ which has been normalized by its degradation rate *k*_4_ just appears in the formulas of *EP*_1_ and *EP*_2_, we define *EP*_*m*_ = *EP*_1_ + *EP*_2_ and use it to represent the energy dissipation in the synthesis-degradation process of ROCK. In the following part, we will study the allocation of total energy dissipation corresponding to different strengthen of external stimulations by the comparison between *EP* and *EP*_*m*_. The Figure S3 shows the trends of the total energy dissipation (i.e., *EP*) and the energy dissipation in the synthesis-degradation process of ROCK (i.e., *EP*_*m*_) with the increase of the strengthen of external stimulations (i.e., *a*) in Mode I and II. It is obviously that their trends are consistent as *a* increases in its respective modes. Specifically, *EP* and *EP*_*m*_ increase simultaneously in Mode I and decrease simultaneously in Mode II with the enhancing of the strengthen of external stimulations (i.e., *a*). Moreover, the difference between *EP* and *EP*_*m*_ diminishes both in Mode I and Mode II when *a* increases. The percentages of energy dissipation in the synthesis-degradation process of ROCK (i.e., *EP*_*m*_) in total energy dissipation (i.e., *EP*) shown in Figures 5C,D are more than 60% both in Mode I and Mode II with different strengthen of external stimulations. In other words, the synthesis-degradation process of ROCK consume more energy than the third state transitions (i.e., *EP*_3_) with different strengthen of external stimulations. Furthermore, Figures 5C,D also show that the percentage of energy which is consumed by the synthesis-degradation process of ROCK increases as the strengthen of external stimulations increases until almost no energy is consumed in process of the third state transitions both in Mode I and Mode II.

## 4. Discussion and Conclusion

In our work, a double-deck loop model is constructed. Due to the stochastic nature of bio-processes (Wang et al., 2017; Yao et al., 2018a,b), we have calculated the steady-state probability distributions of ROCK protein through the method of probability-generating functions for chemical master equations. The crucial factors in the stochastic dynamics of gene switching are identified. It is found that the weights between different pathways (i.e., the internal reaction rates) in DDL are the key point governing the state of gene switching, while an external stimulus fine-tunes this choice preference. Furthermore, the energy consumption in DDL is also discussed. Our results show that most of the energy is required for synthesis and degradation of ROCK, however, a very small amount of energy consumption is required for the basic transition processes of downstream genes between “on” and “off” states. This is because the ROCK processes are not in equilibrium and do not follow detailed balance. But the inter-conversion between “on” state and “off” state is indeed in equilibrium and follows detailed balance. In other words, the two terms in *EP*_3_ defined in Equation (8) cancel each other out because of detailed balance. The theoretical findings about selectivity between different gene states and energy dissipation will be advantageous for our understanding of cell fate determination. Our next steps are to conduct closely related experiments about the development of skeletal muscle and to combine our theoretical study with experimental observations and data.

## Data Availability Statement

The datasets generated for this study are available on request to the corresponding author.

## Author Contributions

QL, MY, and JS conceived and designed the work. QL, JS, FY, LY, YG, and RG carried out computer implementation and theoretical analysis. QL, JS, FY, LY, YG, and RG interpreted the simulation results. MY supervised the project. QL, FY, LY, YG, JS, and RG wrote the original manuscript. QL, MY, and JS contributed to the writing of final manuscript. All authors contributed to the article and approved the submitted version.

## Conflict of Interest

The authors declare that the research was conducted in the absence of any commercial or financial relationships that could be construed as a potential conflict of interest.
