# Supplementary material for: Stochastic Dynamics of Gene Switching and Energy Dissipation for Gene Expression
Source: Front Genet. 2020 Jul 2;11:676. doi: 10.3389/fgene.2020.00676 (PMC7343763; doi:10.3389/fgene.2020.00676)
Supplement: Supplementary file 1 [file Presentation_1.pdf]

Supplementary Information On

Stochastic dynamics of gene switching and energy dissipation for gene expression

Quan Liu [1], FengZhen Yu [2], Liang Yi [3], Yijun Gao [1], Rong Gui [1], Ming Yi [4], Jianqiang Sun [5,*]

[1]Department of Physics, College of Science, Huazhong Agricultural University, Wuhan,China

[2]Department of Chemistry, College of Science, Huazhong Agricultural University, Wuhan,China

[3]College of Animal Science and Technology and College of Veterinary Medicine, Huazhong Agricultural University, Wuhan, China

[4]School of Mathematics and Physics, China University of Geosciences, Wuhan, China

[5]School of Automation and Electrical Engineering, Linyi University, Linyi, China

1 DETAILED DERIVATION FROM CHEMICAL MASTER EQUATIONS TO STEADY-STATE DISTRIBUTIONS

Since it is difficult to obtain the theoretical results of chemical master equations, the method of probability-generating functions is used to change chemical master equations into differential equations which may be solved with mathematical skill. Based on this method, two factorial probability-generating functions $G_i(z) = \sum_{m=0}^{\infty} P_i(m) z^m$ with $i = 0, 1$ are introduced. Substituting these into Eq.2:

$$\begin{aligned} &k_{\text{off}} G_1(z) - k_{\text{on}} G_0(z) + k_2 z G_1'(z) - a k_1 G_0(z) + a k_3 (z-1) G_0(z) - (z-1) G_0'(z) = 0, \\ &-k_{\text{off}} G_1(z) + k_{\text{on}} G_0(z) - k_2 z G_1'(z) + a k_1 G_0(z) + a k_3 (z-1) G_1(z) - (z-1) G_1'(z) = 0. \end{aligned} \quad \text{(S1)}$$

Here, all parameters have been normalized by k_4 as mentioned in Sec.II. It is found from Eq. S1 that

$$(e^{-ak_3 z} G_0)' = -(e^{ak_3 z} G_1)'. \quad \text{(S2)}$$

$e^{-ak_3 z} G_i$ is set as H_i for simplicity. From Eq. S2, we can determine that $H_0' = -H_1'$. This allows us to obtain H_0 from H_1. In the following, we will only focus on the expression of H_1, which can be deduced from Eq. S1 as

$$A(z) H_1'' + B(z) H_1' + C H_1 = 0 \quad \text{(S3)}$$

in which $A(z) = (k_2 + 1)z - 1$, $B(z) = ak_2 k_3 z + k_{\text{off}} + k_{\text{on}} + ak_1 + k_2 + 1$, and $C = ak_2 k_3$. Until now, it seems difficult to obtain the analytical distribution because the coefficients of Eq. S3 are dependent on the variable z. Another transformation of the form $H_1(z) = e^{mz} W(nz + p)$ (here, m, n and p are undetermined constants) is introduced in Eq. S3, and hence a solvable differential equation about W is obtained as

$$\omega W''(\omega) - (\beta - \omega) W'(\omega) - \alpha W(\omega) = 0 \quad \text{(S4)}$$

where

$$\omega = \frac{ak_2k_3}{k_2+1}z - \frac{ak_2k_3}{(k_2+1)^2},$$
$$\alpha = \frac{ak_2k_3}{(k_2+1)^2} + \frac{k_{\text{on}}+k_{\text{off}}+ak_1}{k_2+1},$$
$$\beta = \frac{ak_2k_3}{(k_2+1)^2} + \frac{k_{\text{on}}+k_{\text{off}}+ak_1}{k_2+1} + 1. \quad \text{(S5)}$$

There are two independent solutions of Eq. S4. One is expressed by the Tricomi function of the form $U(\alpha,\beta;\omega)$. This result is inadmissible because it is required that $P_1(m) \to 0$ for $m \to \infty$. The other solution is expressed by the confluent hypergeometric function of the form ${}_1F_1(\alpha,\beta;\omega)$. The analytical expressions for the probability-generating functions are presented as below

$$G_0(z) = A_0[e^{ak_3(z-1)} - e^{ak_3z/(k_2+1)}{}_1F_1(\alpha-1,\beta-1;\omega)],$$
$$G_1(z) = A_0e^{ak_3z/(k_2+1)}{}_1F_1(\alpha,\beta;\omega) \quad \text{(S6)}$$

Furthermore, based on the relationship between probability distribution and generating function:

$$P_i(m) = \frac{1}{m!}\frac{d^m}{dz^m}G_0(z)|_{z=0}. \quad \text{(S7)}$$

The analytic results of probability distributions are obtained and shown in the text (i.e., Eq.3 in Sec.II).

2 THE RESULTS OF NUMERICAL SIMULATIONS

The direct method to verify our analytical results is numerical simulations. The approximate probability distributions of $P_i(m)$ obtained by Monte Carlo simulation are given in Fig.S1.

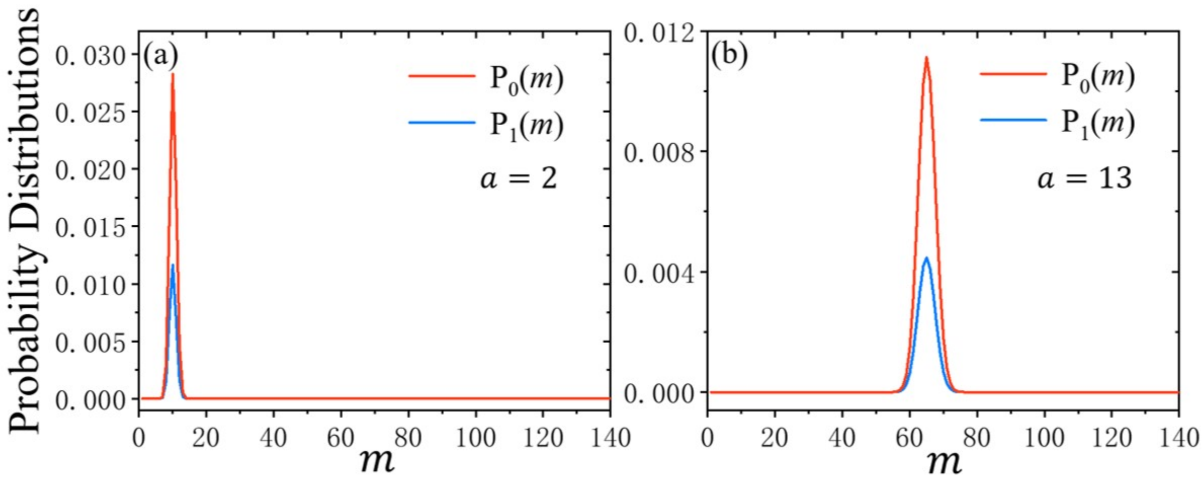

Figure S1. The numerical simulated results about probability distribution functions of the gene's states . The parameters are the same with Fig.3(a) and (b): $k_1 = 20$, $k_2 = 10$, $k_3 = 1.5$, $k_{\text{on}} = k_{\text{off}} = 2$. $P_0(m)$ is the probability of the gene's "off" state indicated with orange curve, $P_1(m)$ is the probability of gene's "on" state indicated with blue curve.

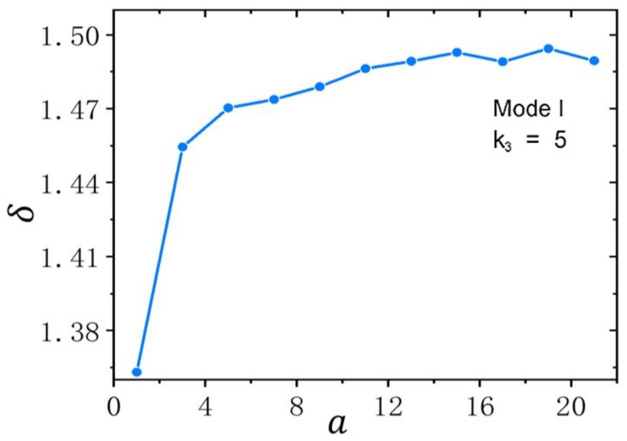

Figure S2. The numerical simulated results about gene's state dominance factor δ which is consistent with that in Fig.4(c). δ will increase with the increasing of the strength of external signal in Mode I. The values of other parameters are listed in Table I.

Moreover, the curve between δ and a which is obtained by Monte Carlo simulation with the same set of parameters in Fig.4(c) is shown in Fig.S2. It is obviously that the trend of δ is similar with the curve in Fig.4(c).

3 THE COMPARISONS BETWEEN THE TOTAL ENERGY DISSIPATION AND THE ENERGY DISSIPATION IN THE SYNTHESIS-DEGRADATION PROCESS OF ROCK IN MODE I AND II

The Fig.S3 shows the trends of the total energy dissipation (i.e., EP) and the energy dissipation in the synthesis-degradation process of ROCK (i.e., EP_m) with the increase of the strengthen of external stimulations (i.e., a) in Mode I and II. It is obviously that their trends are consistent as a increases in its respective modes. Specifically, EP and EP_m increase simultaneously in Mode I and decrease simultaneously in Mode II with the enhancing of the strengthen of external stimulations (i.e., a). Moreover, the difference between EP and EP_m diminishes both in Mode I and Mode II when a increases.

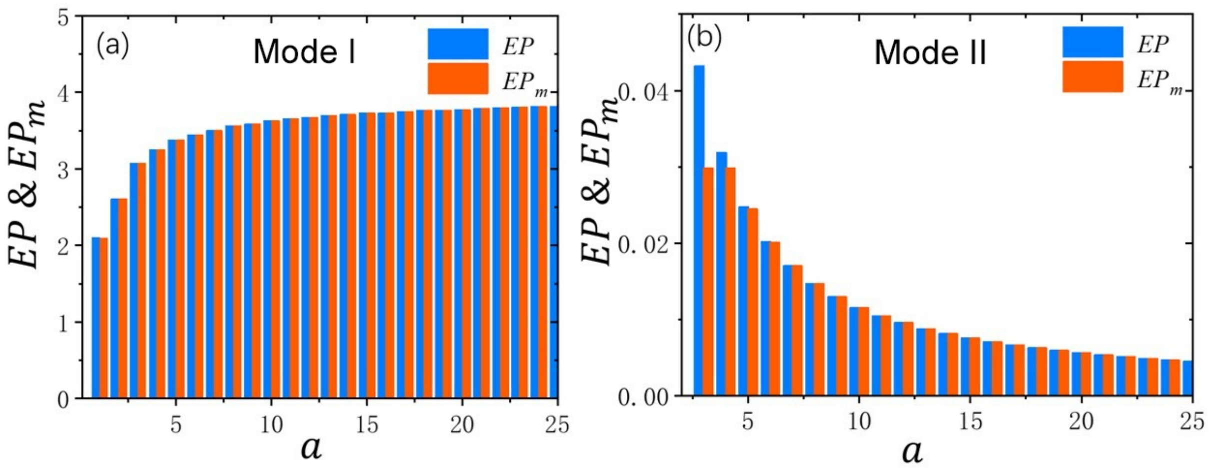

Figure S3. The comparisons between the total energy dissipation and the energy dissipation in the synthesis-degradation process of ROCK in Mode I and II. The values of all parameters are listed in Table I.

4 PARAMETERS TABLE

The values of the main parameters used in calculations are given in Table I. All these parameters have been normalized by the degradation rate of ROCK (i.e., k_4).

Table I: The values of parameters used in calculations

Parameters	Value
The reaction rate of positive control (k_1)	20
The reaction rate of negative control (k_2)	10
The synthetic rate of ROCK (k_3)	1.5 (Mode I) or 5 (Mode II)
The basic switch rate of gene to "on" state (k_{on})	2
The basic switching rate of gene to "off" state (k_{off})	2
